# Awareness and attitude among general dentists and orthodontists toward obstructive sleep apnea in children

**DOI:** 10.3389/fneur.2024.1279362

**Published:** 2024-02-19

**Authors:** Luisa Arcidiacono, Antonio Santagostini, Sara Tagliaferri, Benedetta Ghezzi, Edoardo Manfredi, Marzia Segù

**Affiliations:** ^1^Center of Dental Medicine, University of Parma, Parma, Italy; ^2^Department of Medicine and Surgery, University of Parma, Parma, Italy; ^3^CERT, Center of Excellent Research in Toxicology, University of Parma, Parma, Italy; ^4^IMEM-CNR, Parco Area delle Scienze, Parma, Italy

**Keywords:** sleep, apnea, children, orthodontic, sleep apnea

## Abstract

**Aim:**

This study aimed to investigate Italian dentists’ knowledge of and attitudes toward obstructive sleep apnea (OSA) in children.

**Methods:**

An anonymous questionnaire was prepared using Google Forms and sent to dentists in Italy through private social platforms. The first part of the questionnaire contained basic demographic data questions, and the second part included items about pediatric OSA.

**Results:**

A total of 125 responses were collected within 1 month. The interviews revealed gaps in undergraduate and post-graduate training on OSA, and consequently, low self-evaluation of knowledge and self-confidence in managing young patients with OSA. Dentists showed unfavorable attitudes and poor knowledge of the general findings, risk factors, and consequences of pediatric OSA but demonstrated good knowledge of the beneficial effects of rapid maxillary expansion. Orthodontists showed a more favorable attitude and better recognition of the craniofacial features associated with OSA. In addition, a comparison was made between dentists who had graduated more than 5 years ago and new graduates, and differences were found in undergraduate education, which was better for new graduates, and a small number of questions were better answered by experienced dentists.

**Conclusion:**

This study showed a lack of knowledge about pediatric OSA and its management among Italian dentists, revealing the need to update the dentistry curriculum and organize educational interventions.

## 1 Introduction

Sleep is necessary for health and wellbeing (1–3). It has many functions, including promoting growth, learning, and cognitive development; consolidating memory; enhancing the immune system; and preventing obesity and cardiovascular diseases (1, 2, 4). Special attention must be given to children’s sleep because many childhood lifestyles and conditions have consequences in adulthood (2). According to epidemiological findings in prospective longitudinal studies, poor sleep quality early in life may be associated with emotional and behavioral problems, substance use, and a worse quality of life in adolescents and adults. Moreover, children who sleep well are happier than those with disturbed sleep (5). Sleep-disordered breathing (SDB) may perturb sleep health.

### 1.1 Sleep-disordered breathing in children

SDB comprises a wide clinical spectrum including primary snoring, upper airway resistance syndrome, obstructive hypoventilation, and obstructive sleep apnea (OSA). In primary snoring, which manifests as respiratory noise due to the vibration of the soft tissues (soft palate, uvula, posterior tonsillar pillars) of the oropharyngeal area as air passes through inspiration, alveolar ventilation, blood oxygen saturation, and sleep architecture are maintained at average values. In OSA, partial or complete obstruction of the airways impairs normal ventilation and causes sleep fragmentation (6–8). SDB is common in children; habitual snoring has been reported in 8–12% of children aged 2–8 years, while the prevalence of pediatric OSA has been estimated at about 2–3.5% with two peak periods; the first peak occurs in children aged 2–8 years, while the second peak occurs in adolescents (9). Obstructive sleep apnea syndrome (OSAS) is the most common manifestation of SBD.

### 1.2 Pediatric OSAS

Pediatric OSAS is characterized by the partial or complete obstruction of the airways, which causes cessation or prolonged limitation of airflow during sleep. Impaired ventilation may be associated with oxyhemoglobin desaturation, hypercapnia, and altered sleep patterns (8, 10). OSAS symptoms include snoring, witnessed apneas, nocturnal enuresis, unusual sleeping positions, cyanosis, morning headaches, excessive daytime sleepiness, attention deficit, poor academic performance, hyperactivity, and irritability (9, 11, 12). The pathophysiology of OSA is multifactorial. Pharyngeal muscle physiological hypotonia is associated with non-physiological oropharyngeal space reduction due to abnormal encumbrance of soft tissues and/or craniofacial anomalies, together with altered airflow caused by increased nasal resistance (8, 10, 11). Adenotonsillar hypertrophy is the main risk factor for OSA in children aged 2–8 years, whereas obesity becomes relevant in adolescence. Craniofacial abnormalities, asthma, allergic rhinitis, and syndromic diseases (such as Down syndrome and the Pierre Robin sequence) pose additional risks for the development of OSAS (8, 13). The diagnosis is obtained using validated screening questionnaires and by performing a clinical examination that studies and grades the presence of tonsillar hypertrophy and evaluates craniofacial anomalies, tongue position, and mouth breathing. Moreover, instrumental analysis is required to confirm the diagnosis: Polysomnography is the gold-standard examination for detecting and recording physiological parameters during sleep (oxyhemoglobin saturation, activity in the brain, eye movement, muscular activity in the head and legs, thoracic and abdominal motion, respiratory flow, pulse, body position, actigraphy, and audio recording for detecting snoring). A detected airflow reduction greater than 90% and lasting two respiratory circles or more is considered apnea; in children, a single apnea or hypopnea detected per hour is considered pathological. Polysomnography is the only diagnostic tool that can definitively identify obstructive events and quantify the severity of OSA; however, it is costly and uncomfortable for patients. Hence, especially in children, the clinical diagnosis may be confirmed using night pulse oximetry (8, 12). Early diagnosis and treatment of growing patients are mandatory to prevent severe consequences of the disease. Indeed, OSAS is associated with serious and long-term neurocognitive consequences, behavioral problems, impaired growth, and cardiovascular disease, which can affect the quality of life (7, 11, 14). Awareness and knowledge of OSA are the main barriers to its recognition and appropriate management (15–24). Therefore, adequate training for health practitioners in the field of sleep medicine is important.

### 1.3 Sleep medicine education

Over the past 40 years, there has been an exponential increase in published research on sleep and sleep disorders. However, there exists a slight change in the coverage of sleep in medical school education and textbooks. A study performed in 2006 revealed that major medical textbooks present only few amounts of sleep content, and only a few provide a comprehensive overview of sleep medicine. Moreover, compared to textbook editions from the 1990s, a slight improvement in the attention devoted to sleep was noted in those from the 2000s (3). Similarly, sleep is underrepresented in university curricula. A study on sleep teaching showed that the average amount of time spent on sleep education was just than 2.5 h, with some countries providing more than 3 h and others providing no education. A previous survey of United States medical schools conducted in 1990 reported that a mean of only 2 h was dedicated to sleep and its disorders, or in some schools, education on sleep was not provided. Minimal or no improvement was achieved for 20 years (25). Sleep medicine is not a core educational requirement for several primary specialties, and some neurology residencies and pulmonary fellowships require only a few hours of didactics on sleep, even though these specialists are expected to treat patients with sleep disorders in their practice (4). A survey of how sleep medicine was taught at universities was conducted in Italy. The author, collecting information from 54% of all Italian universities, found that sleep and sleep disorders were taught at 90% of the universities. Nevertheless, in medical school, the average time spent on sleep education was just under 2.5 h against the total of 1,400 h or more of lessons in 6 years. Furthermore, the study showed that in some universities, sleep teaching was organized as an optional didactic activity and that sleep education, when included in the curriculum, was mostly taught during the courses of Neurology, Pneumology, and Dentistry (26). With regard to Dentistry, this result agrees with the pivotal role of dentists in the detection and management of SDB.

### 1.4 The role of the dentist in the screening and management of pediatric OSAS

During clinical examinations, dentists could detect increased palatal vault depth, a large tongue, a high Mallampati score, and other features correlated with OSA. In addition, dental practices are ideal places to complete OSA screening questionnaires, and dentists are health professionals who most frequently see patients; thus, dentists should be sentinels of this disease. Furthermore, oral appliances can be fabricated to treat OSAS (20, 27, 28). When OSA occurs in children, the role of the dentist becomes more crucial. Early diagnosis in growing patients is mandatory to prevent severe and permanent consequences, and dental practice is suitable for OSA screening. Furthermore, orthodontic treatment aimed at correcting craniofacial anomalies, such as rapid maxillary expansion and mandibular or maxillary anterior repositioning devices, could help treat OSA in pediatric patients and prevent its manifestation in adulthood (11, 29, 30). Moreover, there are Italian national guidelines for the prevention and treatment of snoring and sleep apnea syndrome in the developmental age in dentistry which highlight the role of the dentist in detecting snoring and OSAS and intervening therapeutically with the application of oral appliances. According to the guidelines, the dentist must investigate signs and symptoms related to snoring and OSAS and follow a diagnostic and therapeutic pathway characterized by a multidisciplinary approach. Furthermore, it is stated that the dentist, in the case of established snoring or OSAS in a patient of developmental age and in the presence of a related craniofacial morphology, may apply fixed rapid expansion of the maxilla devices and/or mandibular propulsion devices (31). Considering the importance of dentists in managing OSA in young patients, the necessity of adequate knowledge and attitudes among dentists regarding this disease is evident. Furthermore, given the role of orthodontic treatment in the management of pediatric OSA, orthodontists may have more knowledge in this field than general dentists. This study aimed to assess the awareness and attitude of dentists and orthodontists regarding obstructive sleep apnea in children.

## 2 Materials and methods

### 2.1 Study design

A survey was conducted in Italy in February 2023 to assess the knowledge and attitudes among dentists regarding childhood OSA. An anonymous electronic questionnaire was sent through social networks, such as WhatsApp and Facebook, to dentists in Italy. Participants were informed of the study design, their participation was voluntary, and their informed consent was considered to have been obtained by completing the questionnaire.

### 2.2 Study population

Several general dentists and specialist acquaintances of the authors were contacted using WhatsApp or Facebook and were provided a presentation of the study and explained the voluntary nature of participation in the study, ensuring anonymity. Furthermore, the participants were asked to forward the message to other dentists to ensure the dissemination of the questionnaire.

### 2.3 Study instrument

One of the authors developed a questionnaire based on Italian national guidelines for the prevention and treatment of snoring and sleep apnea syndrome in the developmental age and literature. The domains investigated through the questionnaire included the dental curriculum, confidence in management, interdisciplinary approach, referral, risk factors, general findings, consequences, screening, diagnosis, and treatment. The questionnaire was commented upon by other authors and validated by an expert in sleep medicine opinion. The expert reviewed the relevance, readability, comprehensiveness, and clarity of the items. The definitive version of the questionnaire was prepared using Google Forms in the Italian language and included three sections. The first section provided an introduction to the questionnaire and a guarantee of anonymity. In the second section, basic demographic data, such as sex, age, region of provenance, university seat attended, and orthodontic specialization, were collected. The third section included questions on childhood OSA. Specifically, four questions assessed OSAS training, and there were two self-evaluations of knowledge and confidence. One multiple-choice question about the specialists involved in the management of pediatric OSAS and two yes/no questions about referrals were included. Awareness of OSA risk factors in children was evaluated using a Likert-type scale to assess the conditions to which attention was most often paid. Five single-choice or multiple-choice questions assessed knowledge of screening and diagnosis. Moreover, one of the items was a list of conditions, and the respondents were asked to select whether these were consequences of childhood OSA. A similar item indicated knowledge of the positive effects of the rapid expansion of the palate.

### 2.4 Data analysis

Qualitative variables were reported as frequencies, absolute values, or percentages. Polytomous variables were transformed into dichotomous variables for the analysis. Comparisons between groups (general dentists/orthodontists and those graduated more than 5 years ago/less than 5 years ago) were evaluated using the chi-square test or Fisher’s exact test, as appropriate. All statistical decisions were made at a *p-*value of ≤0.05 (two-tailed), and analyses were performed through SPSS Statistics 28 (IBM, Inc., Chicago, IL, USA), whereas Excel was used to prepare graphs.

## 3 Results

### 3.1 Participant demographic characteristics

**Figure 1:**
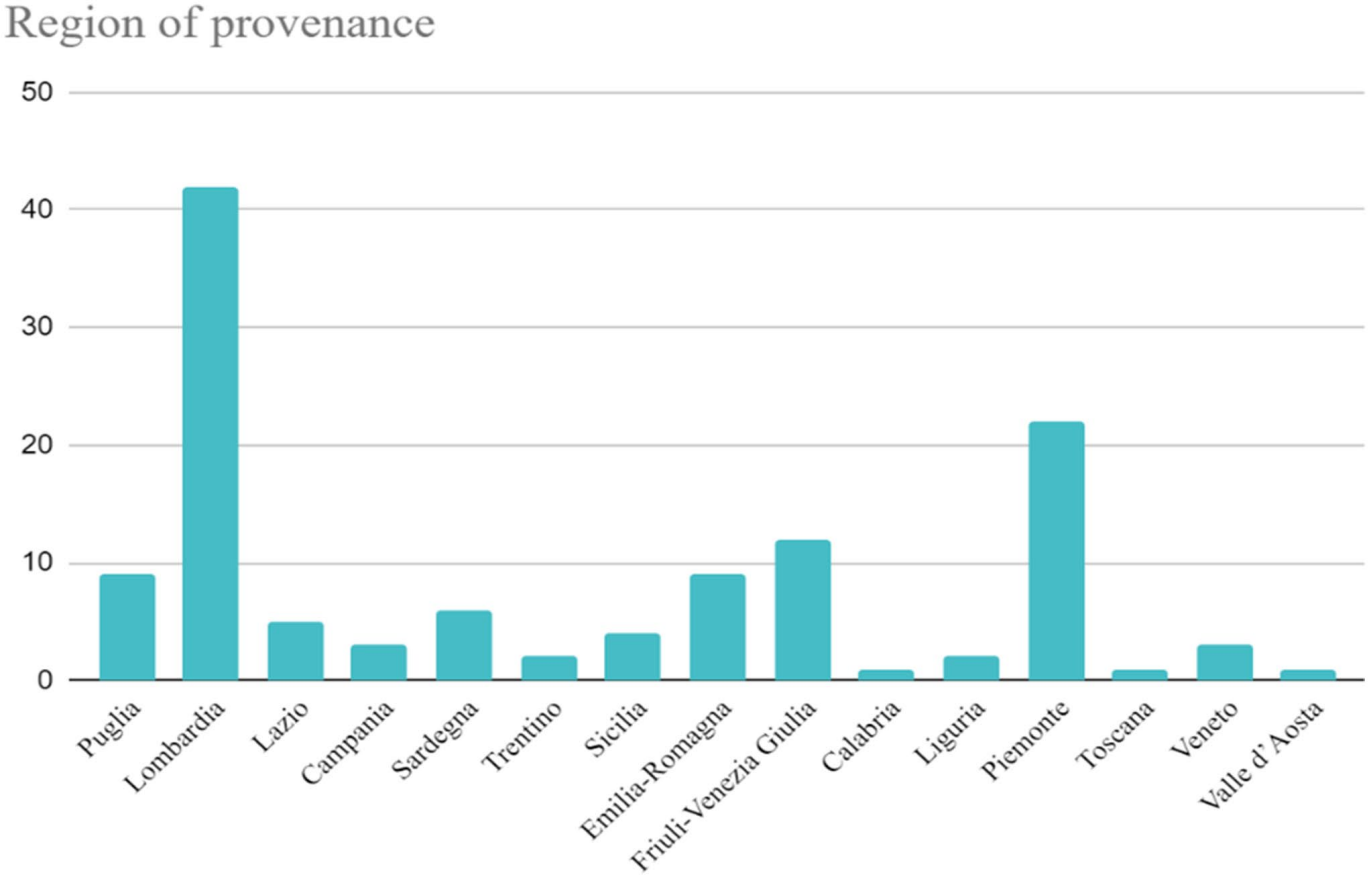
Region of the provenance of the participants.

Within 1 month, a total of 125 responses were collected. The respondents comprised 59 females and 66 males, with a mean age of 47.85 years, ranging from 24 to 71 years. Participants were from different regions of northern, central, and southern Italy (Figure 1), but the majority of them were from Lombardy (42, 33.6%) and Piedmont (22, 17.6%). Moreover, most dentists in this study attended universities in Pavia (45, 36%), Trieste (8, 6.4%), and Parma (7, 5.6%); 9 (7.2%) respondents attended universities abroad. Nearly two-thirds of the participants were general dentists (85.68%), and nearly one-third were orthodontists (40.32%). A total of 106 of the participants had graduated more than 5 years ago (84.8%), while 19 (15.2%) were graduated since less than 5 years.

### 3.2 Training in pediatric OSAS

Approximately two-thirds (86, 68.8%) of the dentists in this study stated that they did not receive any training in pediatric OSA during the degree course, while one-third had received it in the following subjects: orthodontics (17, 13.6%), gnathology (14, 11.2%), and otolaryngology (4, 3.2%). Moreover, approximately half of the respondents (63, 50.4%) answered that they had completed post-graduate education in this field, most through private and continuing medical education (CME) courses (48, 38.4%), and the rest through specialization schools (10, 8%), master’s (3, 2.4%), and other modalities (3, 2.4%).

### 3.3 Self-assessment of pediatric OSA knowledge and confidence in management

**Figure 2:**
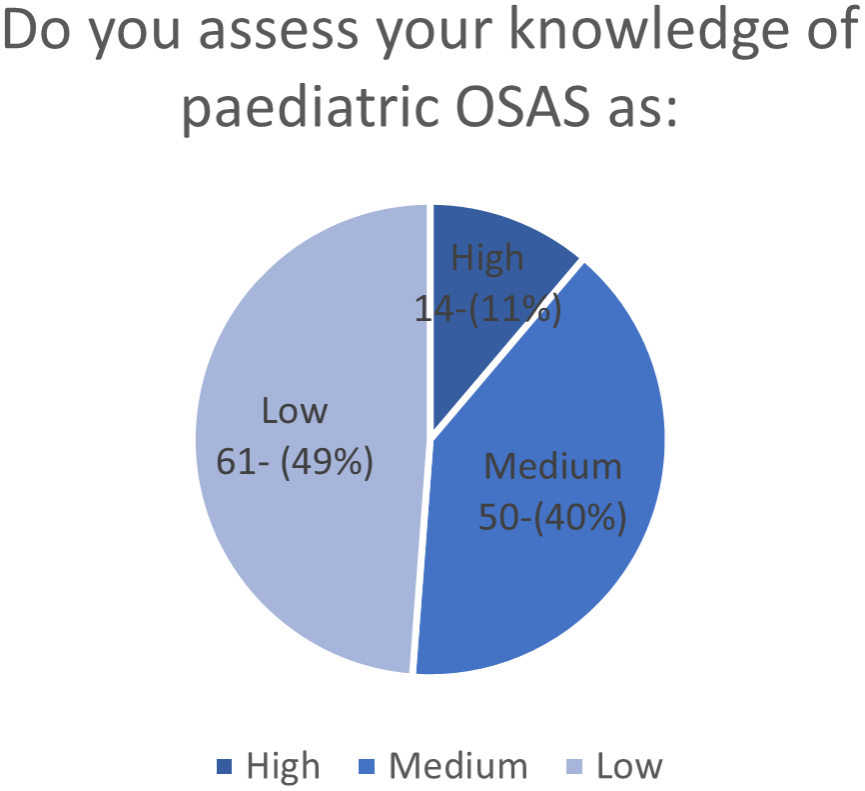
Self-assessment of pediatric OSA knowledge of the participants shown by a pie chart.

Nearly half of the interviewees (61, 48.8%) considered their knowledge of pediatric OSA to be low, and nearly one in ten (14, 11.6%) evaluated their self-knowledge to be high (Figure 2). Furthermore, only half of them (63, 50.4%) felt they had sufficient knowledge to manage children with OSAS.

### 3.4 Multidisciplinary approach

**Figure 3:**
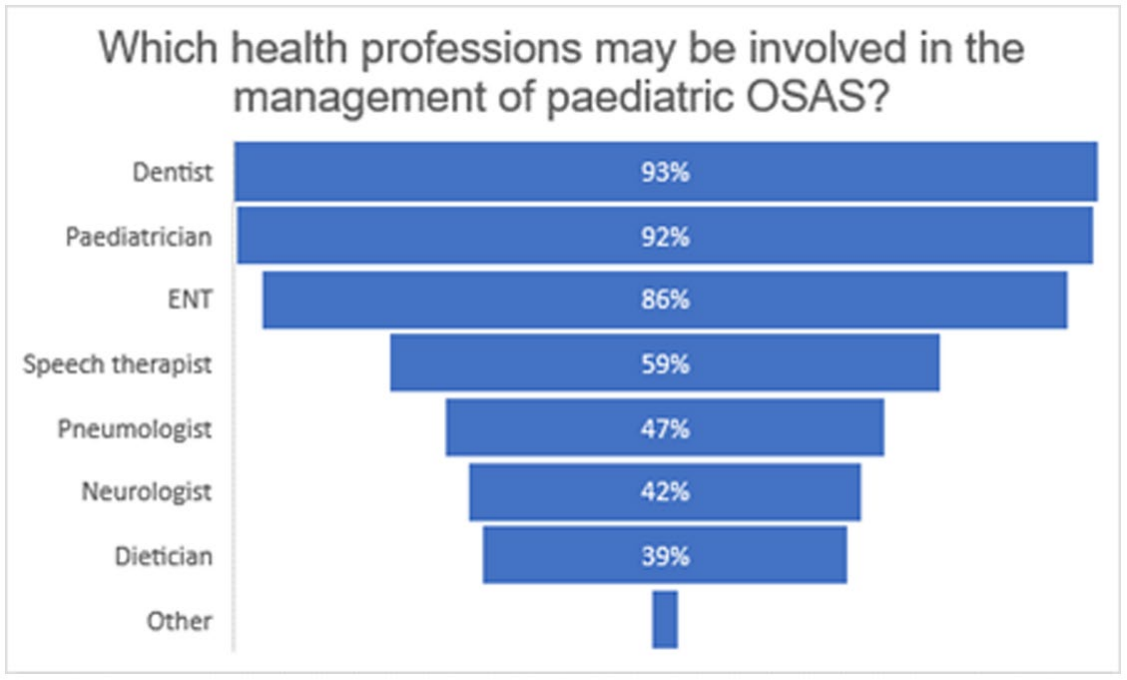
Graph shows the percentages of the different professions listed by the participants as a part of the multidisciplinary team for the diagnosis and management of pediatric OSA. Free answers “more figures” (1 answer) or the identification of other health professionals than those proposed is grouped under ‘other’. The following professions are added (1 answer each): psychologist, exercise science graduate, and osteopath/physiotherapist.

Almost all the dentists involved in this study (115, 92%) believed that the correct management of a young patient with OSAS was achieved through the collaboration of several figures. The majority of respondents included pediatricians (115, 92%) and dentists (116, 92.8%) in potential the multidisciplinary team, whereas less than half felt that pulmonologists (59, 47.2%), neurologists (53, 42.4%), and dieticians (49, 39.2%) should be involved (Figure 3). Although most of the dentists in this study acknowledged their own role in the management of pediatric OSAS, only 57 (45.6%) utilize an orthodontic anamnestic questionnaire which includes questions on signs and symptoms of OSA. Furthermore, despite the recognition of the need for collaboration between different specialists to adequately manage OSAS in children, the referral to other health professionals when OSAS was suspected was found to be low (almost half of the interviewees (71, 56.8%) stated having never referred a patient to other specialists to confirm a diagnosis of OSAS). In addition, only 34 of the respondents (27.2%) stated that they were referred young patients with OSAS for evaluation of orthodontic treatment.

### 3.5 Awareness of risk factors

**Figure 4:**
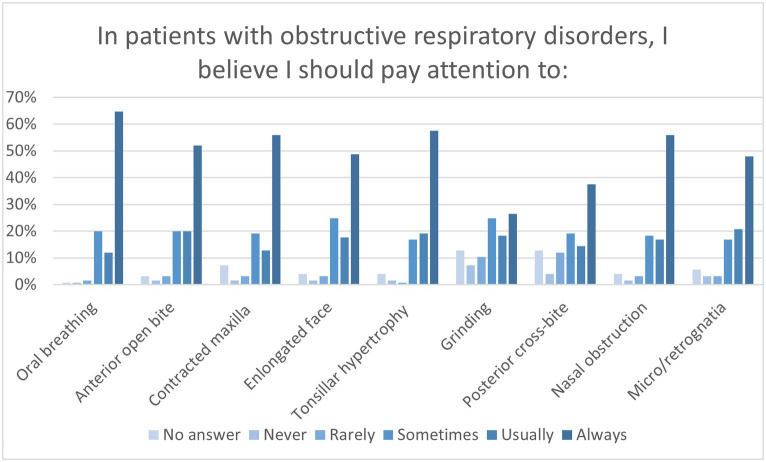
Graph shows the selection percentages of conditions that participants felt they should pay attention to in patients with obstructive respiratory disorders.

Questions investigating the awareness of pediatric OSA risk factors revealed insufficient awareness among dentists regarding some relevant characteristics associated with OSAS in children. In particular, the clinical findings of posterior crossbite, micrognathia, retrognathia, and a history of tooth grinding were underestimated (Figure 4). Furthermore, slightly less than half (53, 42.4%) of the interviewees believed they should not always pay attention to tonsillar hypertrophy in children with obstructive respiratory disorders, and nearly one-third (42, 33.6%) did not consider snoring a warning sign for pediatric OSAS.

### 3.6 General findings

Almost half (59, 47.2%) of the dentists involved in this study were unaware of the existence of national guidelines for the prevention and treatment of obstructive sleep apnea in patients of the developmental age. Moreover, the prevalence of OSA in children was found to be overestimated; only 20% (24) of the interviewees answered correctly when asked to identify the prevalence of the disease, while the majority answered that the prevalence was 10–20% (68, 54.4%) or more (27, 21.6%). Furthermore, only ten (8%) dentists were able to identify the correct value of the apnea–hypopnea index (AHI) that is considered pathological. Therefore, almost all the interviewed (119, 95.2%) acknowledged the proper diagnostic procedure for pediatric OSAS.

### 3.7 Pediatric OSA consequences

**Figure 5:**
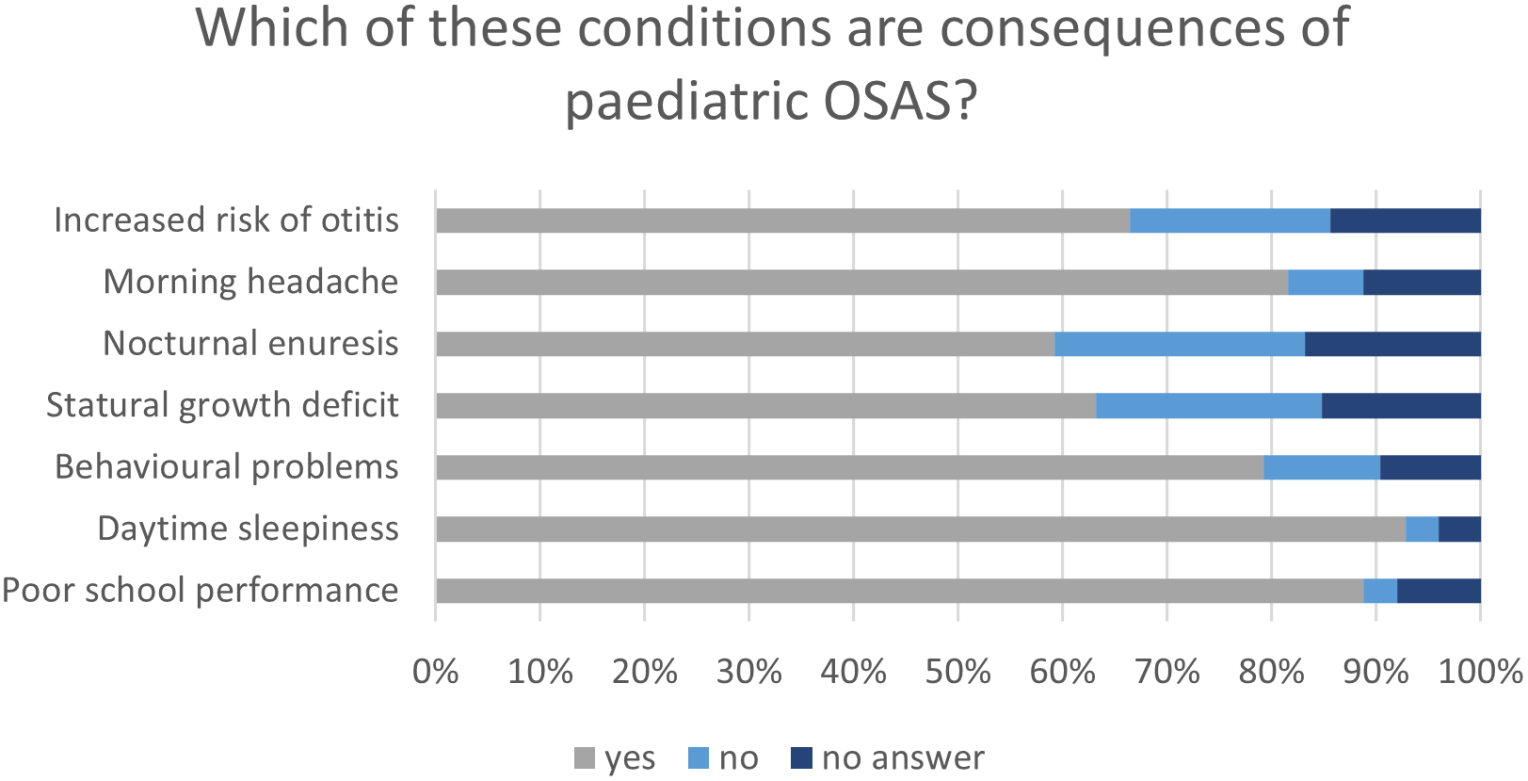
Graph shows the participant’s awareness of the consequences of pediatric OSA.

Dentists were asked to determine whether the following conditions were consequences of OSA in children: poor school performance, daytime sleepiness, behavioral problems, stature growth deficits, nocturnal enuresis, morning headaches, and an increased risk of otitis. Almost all respondents considered daytime sleepiness (116, 92.8%) and poor school performance (111, 88.8%) to be consequences of pediatric OSA; more than two-thirds answered that morning headaches (102, 81.6%), behavioral problems (99, 79.2%), and increased risk of otitis (83, 66.4%) were pediatric OSAS consequences, while less than two-third considered stature growth deficits (79, 63.2%) and nocturnal enuresis (74, 59.2%) as such. Moreover, nearly 20% of the interviewees stated that nocturnal enuresis (30, 24%), stature growth deficits (27, 21.6%), and increased risk of otitis (24, 19.2%) were not consequences of OSA in young patients (Figure 5).

### 3.8 Rapid maxillary expansion effects

**Figure 6:**
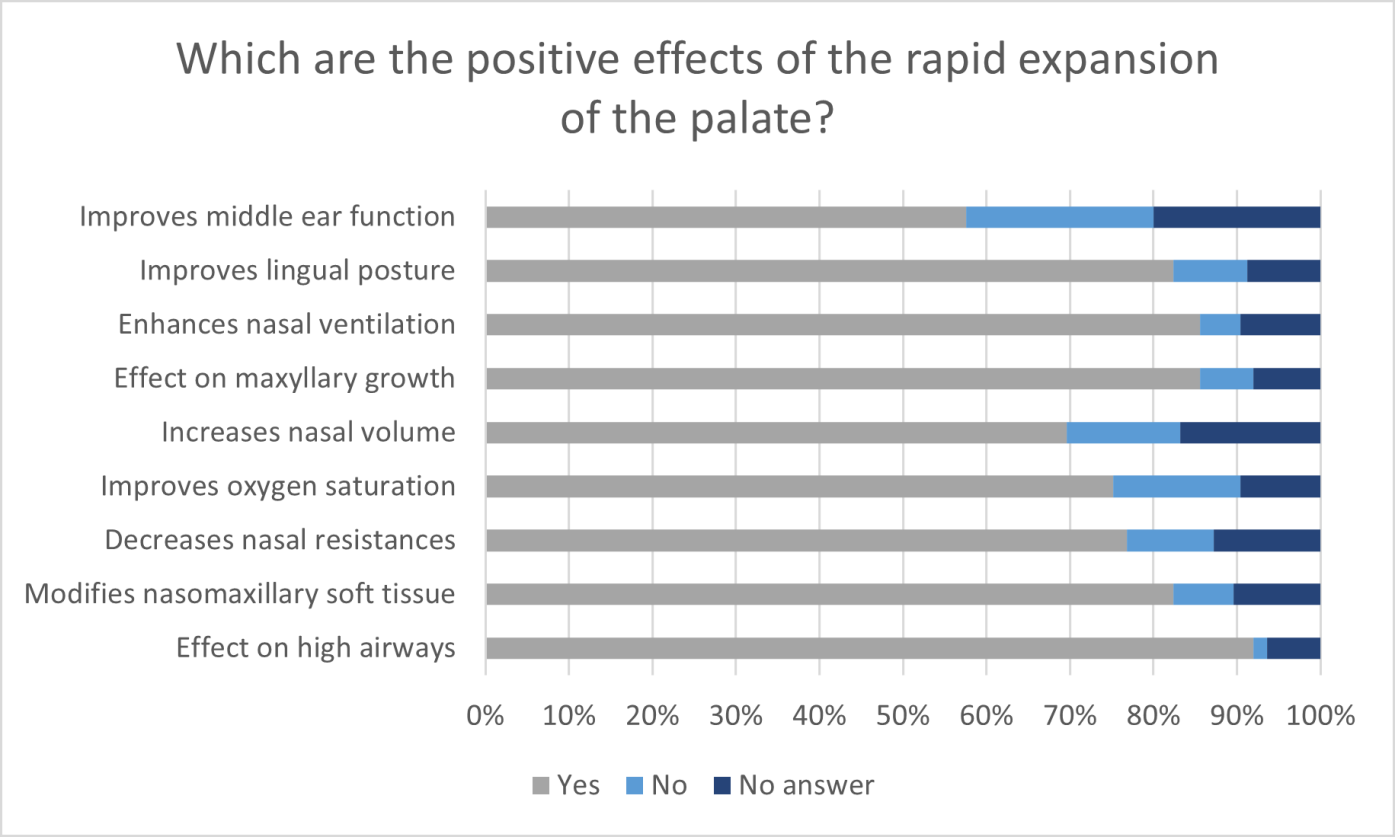
Graph shows the answers to the questions regarding the beneficial effects of rapid maxillary expansion in percentages.

Most of the respondents acknowledged the beneficial effects of rapid maxillary expansion, especially its effect on the upper airways (115, 92%), enhanced maxillary growth (107, 85.6%), and improved nasal ventilation (107, 85.6%). The effects of decreased nasal resistance (96, 76%), improved oxygen saturation (94, 75.2%), and increased nasal volume (87, 69.6%) were only partially recognized. In fact, only two-thirds believed that these were the effects of rapid maxillary expansion, while approximately 10–15% did not consider the same. Moreover, a high number (nearly 10–15%) of dentists did not respond (Figure 6).

### 3.9 Comparison between orthodontist and general dentist

A chi-square test was used to compare the answers provided by orthodontists and those provided by general dentists. Although no significant differences were found between the two groups for most questions, some answers differed significantly. These are shown in Table 1. Moreover, self-assessment of knowledge about OSA in children differed significantly between specialists and general dentists, as shown in Table 2.

**Table 1:** Comparison between orthodontist and general dentist: answers that showed a statistically significant difference.

Questions	General dentists		Orthodontists		*p*-value
	Yes	no	Yes	no	
Have you attended post-graduate training in pediatric OSAS?	42%	58%	68%	33%	*p* = 0.009
Do you think your knowledge of pediatric OSAS allows you to manage a patient within a multidisciplinary team?	46%	54%	65%	35%	*p* = 0.045
Did you include questions regarding the signs and symptoms of OSAS in your orthodontic history questionnaire?	39%	61%	62%	39%	*p* = 0.021
Have you ever sent one of your pediatric patients to a specialist physician to confirm the diagnosis of OSAS?	48%	52%	75%	25%	*p* = 0.005
	Correct	Other	Correct	Other	
In a patient with obstructive respiratory disorders, I believe I should pay attention to contracted upper jaw^*^	69%	31%	89%	11%	*p* = 0.018
In a patient with obstructive respiratory disorders, I believe I should pay attention to posterior crossbite^*^	52%	48%	83%	17%	*p* = 0.002

^*^Polytomous variables. The answers were transformed into dichotomic variables: “always” and “usually” were grouped as “correct,” while other possible answers were grouped as “other”.

**Table 2:** Comparison between orthodontist and general dentist: self-assessment of knowledge about OSA in children.

		Do you feel your pediatric OSAS knowledge is:						Total	%
		Low		Medium		High			
		*N*	%	*N*	%	*N*	%	*N*	
Did you specialize in orthodontics?	No	50	82.0%	26	54.2%	7	50.0%	83	67.5%
	Yes	11	18.0%	22	45.8%	7	50.0%	40	32.5%
Total		61	100.0%	48	100.0%	14	100.0%	123.100%	

	Value	df	Asymptotic Significance (2-sided)
Pearson Chi-Square	11,660^a^	2	0.003

The table shows the frequencies and percentages of respondents who rated their knowledge as low, medium, or high, separating specialists in orthodontics and general dentists. The result of the chi-square test is a *p-*value = 0.003.

### 3.10 Comparison between fresh graduates and graduates from over 5 years ago

Another comparison was conducted between dentists who had graduated more than 5 years ago and those who had graduated less than 5 years ago using the chi-square test. Answers showing statistically significant differences are presented in Table 3. However, most questions were similarly answered by both groups.

**Table 3:** Comparison between dentists who had graduated more than 5 years ago and those who had graduated less than 5 years ago: answers that showed a statistically significant difference.

Questions	<5 years		>5 years		*p*-value
	Yes	no	Yes	no	
Did you receive pediatric OSAS training during your degree course?	63%	37%	26%	74%	*p* = 0.001
Have you ever sent one of your pediatric patients to a specialist physician to confirm the diagnosis of OSAS?	32%	68%	61%	39%	*p* = 0.016
Should snoring in children always be considered an alert bell regardless of the diagnosis of OSAS?	32%	68%	72%	28%	*p* < 0.001
Does rapid maxillary expansion improve middle ear function?	53%	47%	72%	28%	*p* = 0.048
	“all of the above”	Other	“all of the above”	Other	
What is the diagnostic pathway for pediatric OSAS?^*^	84%	16%	97%	3%	*p* = 0.045

^*^Polytomous variables. The answers were transformed into dichotomic variables creating two groups: “all of the above” (correct answer) and “other,” which enclosed the other possible answers. The *p*-value of were obtained using Fisher’s exact test.

## 4 Discussion

Only a few studies have assessed knowledge and attitudes toward OSAS among dentists, and even fewer have been concerned with pediatric OSAS. This study aimed to evaluate Italian dentists’ awareness of and attitudes toward OSA among children. In the current study, dentists from different regions of Italy were involved; nevertheless, a large number of interviewees were from Lombardy and Piedmont; thus, the population of the study could not be representative of Italian dentists.

### 4.1 Training in pediatric OSA

The results showed that only one-third of the interviewed participants had received training in pediatric OSAS during the degree course. Similarly, in a study by Alharbi et al. (32), approximately half of the participants (45, 4%) were unaware if their undergraduate curriculum included information on pediatric OSA. Therefore, in this study, this apparent conflict could be partly explained by considering the age range of the sample and the recently increasing interest in sleep medicine. In support of this, it can be noted that among those who claimed to have received OSAS training, there was a statistically significant difference between those who had graduated more than 5 years ago and those who had graduated for a lesser duration (*p* = 0.001). Thus, although the results seem to indicate that there are serious deficiencies in university education in pediatric OSAS, the data likely overestimate the actual gaps. Moreover, the results showed that pediatric OSAS is mainly taught during the course of orthodontics and gnathology, highlighting the pivotal role of orthodontists in managing young patients with OSAS. Approximately half of the respondents (63, 50.4%) had undertaken post-graduate education in pediatric OSA, most through private and continuing medical education (CME) courses (48, 38.4%) and the rest through specialization school (10, 8%), master’s (3, 2.4%), and other modalities including self-taught approaches (3, 2.4%). Differences were found in the results obtained in a recent study by Alzahrani et al. (20) in which the major source of previous knowledge about OSA among interviewed dentists was dental school (39.8%). Moreover, the self-taught approach (28%) was mentioned by a greater number of dentists than in our study (1, 0.8%) as a source of information regarding OSA, while the percentages of answers mentioning post-graduate training (22%) and conferences (18.8%) were similar between the two. These differences could be because the previously cited study did not investigate knowledge in the specific field of pediatric OSAS. Data on pediatric OSAS training generally indicate inadequate education in this field. Thus, the fact that in our study, only 11.6% of the dentists involved in the search believed they had high knowledge and that only half of the interviewees (50.4%) thought they could handle young patients with OSA is not surprising, although this highlights the need for improving pediatric OSA education. Lower percentages of self-confidence in managing OSA in children were found among physicians (29.2%) in a study by Uong et al. (19) and among medical students in a study by Wadhwa et al. (33).

### 4.2 Multidisciplinary approach

A multidisciplinary approach was deemed important to correctly manage pediatric OSA by almost all the dentists involved in this study (92%). Most believed that pediatricians (92%) and ENT (86%) should be involved in the management of the disease, while nearly half or more did not include speech therapists, pneumologists, neurologists, and dieticians in the potential multidisciplinary team. Moreover, despite recognizing the importance of collaborating with other specialists, more than half (56.8%) had seldom referred a patient to other specialists to confirm the diagnosis of OSAS. Similar results were reported by Alzahrani et al.; 84.4% of the dentists interviewed recognized the importance of referring to physicians when OSAS was suspected but only 14% knew the protocol to refer the sleep medicine department in their workplace (20). In a study by Alrejaye et al., physicians showed less than favorable attitudes toward the role of dentists in the management of OSAS, and only 23.5 and 12.8% of the physicians, respectively, involved in the study believed they should usually and always refer patients diagnosed with OSAS to dentists for a more comprehensive assessment (34). These results agree with the findings of this study, according to which only 27.2% of dentists stated they were referred young patients with OSAS for orthodontic treatment evaluation.

### 4.3 Awareness and knowledge of symptoms and risk factors

The role of dentists in the management of young patients with OSA is pivotal, but even more crucial is their role in identifying the disease. Answers to questions about risk factors for pediatric OSA showed that dentists paid more attention to characteristics of the adenoidal phenotype (mouth breathing, contracted maxilla, anterior open bite, elongated face, tonsillar hypertrophy, and nasal obstruction), even if only half of the interviewees usually or always paid attention to posterior crossbite, while micrognathia and retrognathia were underestimated. Moreover, insufficient attention has been paid to nighttime symptoms, such as grinding and snoring. It is interesting to note that in our study, 20% of the dentists involved claimed they did not usually attend to hypertrophic tonsils, while in the study by Alharbi et al., 96.2% of dentists and 97% of physicians correctly identified it as a risk factor for OSA in children, and likewise in other studies by Uong et al. and Tamay et al., 85.9 and 95% of physicians, respectively, believed that adenotonsillar hypertrophy was the most frequent contributing factor for OSA in young patients (19, 32, 35). Furthermore, Alharbi et al. found that only 21.4% of the dentists and 23.6% of the physicians were informed of the prevalence of OSA in children, similar to the results of this study (20%). However, in the study by Uong et al., a higher number of physicians answered correctly to the question about prevalence (55.1%) and even more in the study by Tamay et al. (62%) (19, 32, 35). In our study, most dentists overestimated the prevalence of OSA in children, answering that it was 10–20% or more, and 30.4% of the interviewees answered that the pathological value of apnea and hypopnea index in children was >5. These answers would be correct if the questions referred to OSA in adults; thus, these mistakes were likely due to better knowledge of adult OSAS than OSA in children, as seen in the study by Chang et al. (17). In our study, dentists demonstrated poor awareness of nocturnal enuresis, stature growth deficits, and increased risk of otitis as consequences of OSA in children, while poor school performance and behavioral problems were identified as consequences of pediatric OSA by most dentists, in disagreement with the findings of Alharbi et al. and Wadhwa et al., where questions on these topics were poorly answered (32, 33).

### 4.4 Knowledge of beneficial effects of rapid maxillary expansion

To our knowledge, this is the first study assessing dentists’ knowledge of rapid maxillary expansion. The results showed an overall decent knowledge about its beneficial effects.

### 4.5 Comparison between orthodontists and general dentists

In pediatric OSA, orthodontists might show better knowledge and attitudes because they are likely more involved in the identification and management of the disease than general dentists. Indeed, they see young patients at the ages corresponding to the two peaks of prevalence of OSA in children and provide oral appliances to correct craniofacial abnormalities, which may be predisposing factors for OSA. We were interested in whether there were differences between orthodontists’ and general dentists’ knowledge of and attitudes toward pediatric OSA. A previous study by Vuorjoki-Ranta et al. showed that specialists had better knowledge of the consequences, symptoms, and management of OSAS than general dentists, likely due to a more advanced educational level. Specialists were better able to recognize signs and symptoms of OSA in children and were more prone to examine factors related to OSA (36). In our study, we compared the answers of general dentists and orthodontists and found no statistically significant differences for most questions. Nevertheless, it would be interesting to take a closer look at the questions to which general dentists and orthodontists answered differently. Orthodontists had more post-graduate training in pediatric OSA than general dentists (*p* = 0.009). This was partly achieved through specialization, mostly through private courses and continuing medical education; thus, it reflects a greater interest in orthodontists than general dentists in children with OSA. Furthermore, more orthodontists addressed questions regarding the signs and symptoms of OSAS in the orthodontic history questionnaire (*p* = 0.021). Orthodontists showed a higher self-evaluated level of knowledge (*p* = 0.003) and better self-confidence in managing young patients with OSA, although this difference was not statistically significant (*p* = 0.045). Referrals to physicians showed a highly significant difference (*p* = 0.005) between orthodontists and general dentists. Vuorjoki-Ranta et al. found that consultation was reported as frequent for specialists and dentists in their late careers likely because they had obtained more possible contacts for referral (36). In our study, general dentists and freshly graduated dentists referred their patients to physicians when OSA was suspected less often than orthodontists and dentists who graduated more than 5 years ago (*p* = 0.016). Furthermore, orthodontists paid more attention to craniofacial characteristics associated with OSA, especially posterior crossbite (*p* = 0.002) and contracted upper jaw (*p* = 0.018), as expected, considering their more advanced education and higher interest in craniofacial features.

### 4.6 Comparison between fresh graduates and dentists graduated more than 5 years ago

Along with the comparison between general dentists and orthodontists, we were interested in whether there were differences in the knowledge and attitude of pediatric OSA between fresh graduates and those graduated more than 5 years ago. Any differences between these groups could be the result of a greater clinical experience but could also be related to the increased attention paid to pediatric OSA since the 2000s, whereby those graduated for less than 5 years might have received better training in this field. In fact, this comparison showed that more dentists graduated less than 5 years ago received undergraduate education (*p* = 0.001); however, it is alarming to notice that 37% of the fresh graduates did not receive any pediatric OSA education during undergraduate courses. Fresh graduates performed worse than experienced dentists in terms of the frequency of referral to physicians, as seen before, and when considering snoring as a warning sign in children (*p* < 0.001). Moreover, even if there was no strong statistical significance, differences were found in recognizing the beneficial effect of rapid maxillary expansion on middle ear function (*p* = 0.048) and in following the correct diagnostic pathway for pediatric OSA (*p* = 0.045).

## 5 Conclusion

Despite the increasing awareness of the role of dentists in screening young patients for OSA and managing the disease, training in pediatric OSA is still disregarded in the undergraduate dental curriculum. Thus, education in this field is mainly achieved through private courses and continuing medical education and is left to self-interest. The result of this system of training is low self-confidence in dentists in managing the disease, associated with gaps in knowledge and unfavorable attitudes. Orthodontists play a crucial role in managing young patients with OSA. Nevertheless, despite having a more advanced education level, they did not show markedly better knowledge and attitudes than general dentists in our study. Improving undergraduate education and organizing educational interventions for pediatric OSA should increase dentists’ confidence in identifying and managing young patients with OSA, leading to earlier diagnosis, proper referral, treatment based on a multidisciplinary approach, reduced risk of complications, and hence better quality of life for patients. Further studies are needed to evaluate the efficacy of improvement in pediatric OSA education among dentists, preferably conducted using a single validated questionnaire designed for dentists to compare the results of different studies.

## Data availability statement

The raw data supporting the conclusions of this article will be made available by the authors, without undue reservation.

## Ethics statement

Ethical approval was not required for the studies involving humans because this is a study on the attitude of dentists rather than an experimental study. The studies were conducted in accordance with the local legislation and institutional requirements. The participants provided their written informed consent to participate in this study.

## Author contributions

LA: Investigation, Writing – original draft. AS: Methodology, Writing – review & editing. ST: Data curation, Writing – review & editing. BG: Writing – review & editing, Investigation. EM: Investigation, Writing – review & editing. MS: Writing – review & editing, Conceptualization.
